# Beyond Computer-Aided Diagnosis: Artificial Intelligence as a “Digital Mentor” for POCUS Image Acquisition and Quality Assurance: A Narrative Review

**DOI:** 10.3390/diagnostics16060858

**Published:** 2026-03-13

**Authors:** Hyub Huh, Jeong Jun Park

**Affiliations:** 1 Department of Anesthesiology and Pain Medicine, Kyung Hee University Hospital at Gangdong, Kyung Hee University College of Medicine, Seoul 05278, Republic of Korea; clumania@gmail.com; 2 Department of Anesthesiology and Pain Medicine, CHA Bundang Medical Centre, CHA University School of Medicine, Seongnam 13496, Republic of Korea

**Keywords:** clinical governance, digital mentor, free open access medical education, point-of-care ultrasound, quality assurance, ultrasound education

## Abstract

Point-of-care ultrasound (POCUS) is portable and radiation-free, but its clinical reliability is constrained by operator-dependent image acquisition and the limited scalability of expert quality assurance (QA) review. As handheld devices proliferate faster than mentorship capacity, trainees increasingly rely on heterogeneous free open access medical education (FOAMed) resources that rarely provide real-time psychomotor feedback. We conducted a structured narrative review (MEDLINE, Embase, Scopus, and Web of Science; last searched on 23 February 2026), with searches performed by H.H. and independently checked by J.J.P. (both POCUS-trained clinicians). After screening, 31 studies were included. We synthesized evidence on artificial intelligence (AI) systems that support bedside image acquisition and automate QA. The primary synthesis centered on key prospective or comparative clinical evaluations of AI-guided acquisition across echocardiography, focused assessment with sonography in trauma, abdominal aortic aneurysm screening, and lung ultrasound, complemented by peer-reviewed studies of FOAMed appraisal tools and online resource quality. These evaluations suggest that real-time probe guidance, view recognition, anatomy labeling, and automated capture may enable novices, after brief training, to acquire diagnostically adequate images for narrowly defined tasks. Early reports of automated QA scoring and program-level triage for expert review suggest potential to reduce expert workload and shorten feedback cycles, but external validation, generalizability across devices and patient habitus, and patient-centered outcomes remain limited. Acquisition-focused AI may therefore serve as an upstream “digital mentor” to improve novice image acquisition. We propose a practical pathway that integrates curated FOAMed resources and simulation with AI-guided bedside acquisition and continuous QA governance for safe deployment.

## 1. Introduction

### 1.1. Point-of-Care Ultrasound and Modern Bedside Diagnostics

Point-of-care ultrasound (POCUS) is increasingly used for bedside decision-making because it provides rapid, repeatable imaging without ionizing radiation [1,2]. These strengths are particularly valuable in time-sensitive settings where reassessment is frequent. In contrast, computed tomography (CT) provides broad anatomic detail and diagnostic coverage but typically requires transport and fixed infrastructure and exposes patients to ionizing radiation [3]. The availability of handheld devices has lowered barriers to ultrasound deployment across emergency medicine, critical care, internal medicine, anesthesiology, and cardiology [4].

However, unlike CT, ultrasound remains highly operator-dependent at acquisition [5,6]. When the target view is not obtained, neither expert interpretation nor downstream algorithms can recover missing information. For many bedside failures, the limiting step is therefore acquisition rather than interpretation.

### 1.2. The Operator Gap and the Hardware–Humanware Mismatch

As devices diffuse faster than training capacity, programs face a persistent “hardware–humanware” mismatch: the technology is available, but consistent acquisition skill is not. Current training models still rely on apprenticeship, intermittent bedside mentorship, and specialty-specific curricula [7,8]. Ultrasound acquisition is a psychomotor task that requires continuous micro-adjustments in probe position, orientation, pressure, and insonation angle, and learning curves are variable and often prolonged [5]. Competency models based only on scan counts are therefore unreliable. Common failure modes include incomplete protocols, suboptimal settings, and artifact mismanagement; these errors can persist when feedback is absent [6].

### 1.3. Informal Learning and FOAMed: Accessibility Without Psychomotor Feedback

In settings where local mentorship is limited, trainees often turn to informal, self-directed learning through free open access medical education (FOAMed) resources [9,10]. Accordingly, structured appraisal and curation tools (e.g., METRIQ/rMETRIQ and AIR) are important to mitigate variability in content quality [11,12,13,14].

Yet, evaluations of widely available video-based instructional materials demonstrate heterogeneous quality and incomplete coverage of key steps [15]. Even high-quality resources largely provide cognitive scaffolding; they rarely deliver closed-loop, individualized feedback on probe motion and image adequacy at the bedside.

### 1.4. Reframing Artificial Intelligence (AI) as an Upstream “Digital Mentor” for Acquisition and Quality Assurance (QA)

Most ultrasound AI research has emphasized interpretation and computer-aided diagnosis [16,17,18,19]. This emphasis does not address a frequent bedside failure mode: inability to obtain diagnostic-quality images in the first place. Acquisition-focused AI aims to operate upstream by guiding probe motion, confirming target views, and assessing adequacy during scanning, while automated QA extends this logic to post-scan review and program-level feedback loops [20,21,22,23,24,25,26].

Prospective and comparative evaluations in echocardiography, focused assessment with sonography in trauma, abdominal aortic aneurysm screening, and lung ultrasound suggest that real-time guidance, view recognition, anatomy labeling, and automated capture can help novices obtain diagnostically adequate images for prespecified tasks after brief training [20,21,22,23,24,25]. Early work also suggests that automated QA scoring and triage may reduce expert review burden, but patient-facing outcomes and external generalizability remain limited [26].

**Figure 1:**
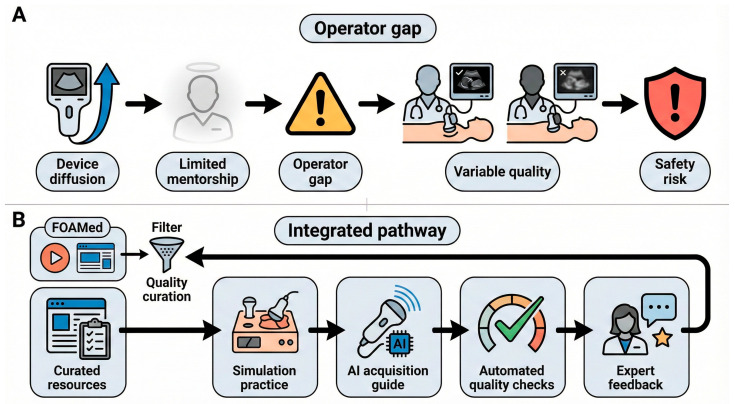
Operator gap and an integrated pathway for point-of-care ultrasound training and governance (conceptual framework). (**A**) illustrates the operator gap when device diffusion outpaces mentorship, leading to variable acquisition quality and safety risk. (**B**) outlines an integrated pathway: curated resources and simulation for fundamentals, AI-guided bedside acquisition, and automated quality checks plus expert feedback for QA within governance. This framework is conceptual and is informed by the evidence synthesized in this review and existing training and governance recommendations.

Accordingly, this review intentionally shifts the center of gravity from “AI as an interpreter” to “AI as a digital mentor” for acquisition and scalable QA. We synthesize evidence on acquisition-support and QA functions and propose a pragmatic pathway that integrates curated FOAMed, simulation, AI-guided bedside acquisition, and continuous QA within governance structures (Figure 1) [1,2,6].

## 2. Methods

Narrative reviews are vulnerable to selection bias when evidence identification is implicit. To strengthen methodological transparency, we conducted a structured narrative review with explicit scope definitions, a structured database search strategy, source prioritization rules, and reporting checks. We did not perform a full systematic review or meta-analysis because the available literature is heterogeneous in study designs, AI functions, protocols, reference standards, and endpoints, limiting meaningful quantitative pooling. The full search strategies are provided in Supplementary File S1 to support reproducibility and updating. Reporting was guided by the Scale for the Assessment of Narrative Review Articles (SANRA) [27], and the completed SANRA checklist is provided as Supplementary File S2. A domain-based qualitative risk of bias appraisal (informed by QUADAS-2) is provided as Supplementary File S3, focusing on representative early clinical evaluation and QA program studies.

### 2.1. Scope and Key Definitions

We defined three domains a priori: (1) AI-assisted image acquisition, defined as AI functions intended to improve operator performance during scanning (e.g., view recognition, anatomy labeling for orientation, prescriptive probe guidance, and automated capture triggers); (2) automated QA, defined as AI functions intended to assess image adequacy, completeness, or protocol adherence during or after scanning and to support QA processes at the individual and program level (we distinguished real-time adequacy assessment from program-level workflow tools in the taxonomy below); and (3) the learning ecosystem and governance domain, which included FOAMed, informal self-directed learning, and structured appraisal and curation tools.

In this review, we use the term “digital mentor” operationally to describe operator-facing AI systems that provide real-time or near-real-time, actionable feedback during scanning (e.g., probe motion cues, view confirmation, adequacy scoring with confidence communication, or automated capture) with the explicit goal of helping a learner obtain diagnostically adequate images and linking acquisition events to a QA feedback loop. We use this term to distinguish these systems from post-acquisition interpretive decision support tools that do not intervene in the acquisition process or quality governance.

### 2.2. Literature Identification and Sources

We searched MEDLINE (PubMed), Embase, Scopus, and Web of Science Core Collection using two a priori concept sets: (A) artificial intelligence-assisted ultrasound image acquisition and automated quality assurance, and (B) FOAMed and online ultrasound learning quality appraisal. The searches were initially executed on 6 February 2026 and updated on 23 February 2026 (final search date); no formal pilot searches were performed beyond iterative refinement of keywords and database-specific syntax during protocol development. Searches were conducted by H.H. and independently checked by J.J.P. for completeness. Both authors are POCUS-trained clinicians.

**Figure 2:**
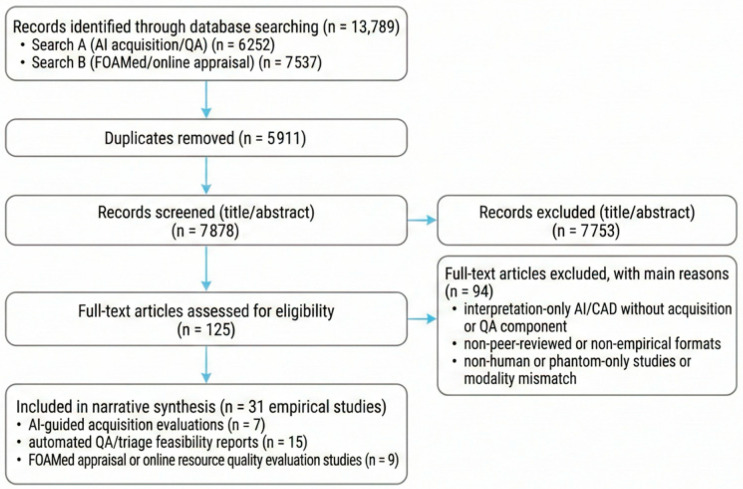
PRISMA-lite study identification and selection workflow for this structured narrative review.

Records were screened at the title/abstract and full-text levels for relevance to acquisition-focused AI, automated QA, and peer-reviewed FOAMed appraisal in ultrasound education. We included peer-reviewed human studies evaluating AI functions intended to improve image acquisition (e.g., real-time guidance, view recognition, anatomy labeling, automated capture) or to automate QA/triage of scan quality, as well as peer-reviewed studies that evaluated FOAMed quality or described appraisal instruments (e.g., METRIQ/rMETRIQ and AIR). We excluded papers focused solely on interpretive computer-aided diagnosis without acquisition or QA components and non-peer-reviewed online posts or videos. Disagreements were resolved by discussion and consensus between the two authors. The workflow is summarized in a PRISMA-lite study flow diagram (Figure 2). In brief, across both searches, we identified 13,789 records, removed 5911 duplicates, screened 7878 titles/abstracts, assessed 125 full texts, and included 31 empirical studies.

We prioritized peer-reviewed research and did not systematically review FOAMed posts or videos; instead, we relied on peer-reviewed evaluations and appraisal instruments. Because online content changes rapidly, direct enumeration of FOAMed posts would be difficult to reproduce.

### 2.3. Evidence Weighting and Synthesis Approach

We prioritized prospective clinical evaluations and comparative, multicenter studies, with explicit reference standards for diagnostic adequacy (e.g., masked expert review). We summarized evidence by clinical domain, operator experience, device integration, AI function, and reported outcomes, and used narrative synthesis to link evidence clusters to training and governance implications.

### 2.4. Reporting and Evaluation Checks

We extracted methodological features relevant to clinical translation, including cohort spectrum, reference standard, external validation, device diversity, and failure modes. We mapped reporting elements to established guidance when applicable, including the Consolidated Standards of Reporting Trials extension for Artificial Intelligence (CONSORT-AI), the Standard Protocol Items Recommendations for Interventional Trials extension for Artificial Intelligence (SPIRIT-AI), the Developmental and Exploratory Clinical Investigations of Decision support systems driven by Artificial Intelligence (DECIDE-AI), the Transparent Reporting of a multivariable prediction model for Individual Prognosis Or Diagnosis extension for Artificial Intelligence (TRIPOD+AI), and the Standards for Reporting Diagnostic Accuracy Studies 2015 (STARD 2015) and Standards for Reporting Diagnostic Accuracy Studies for Artificial Intelligence (STARD-AI) [28,29,30,31,32,33]. For imaging-specific transparency, we referenced the Checklist for Artificial Intelligence in Medical Imaging (CLAIM) [34]. When interpreting diagnostic accuracy style evaluations, we considered concepts from the Quality Assessment of Diagnostic Accuracy Studies 2 (QUADAS-2) tool [35]. These instruments were used as qualitative prompts for reporting appraisal rather than as formal scoring tools.

### 2.5. Qualitative Appraisal of Common Methodological Limitations

Across included early-phase evaluations, common limitations included constrained protocols and narrowly defined intended uses, frequent single-center and single-device designs, limited reporting of consecutive enrollment and patient spectrum (spectrum bias), and reliance on expert-rated image adequacy as a surrogate endpoint. Several studies were performed in laboratory or feasibility contexts with small samples, and operator learning curves may confound short-term performance gains. External validation across devices, sites, and patient body habitus was uncommon, and few studies reported patient-centered outcomes, cost-effectiveness, or post-deployment monitoring. Accordingly, reported improvements in acquisition quality should be interpreted as feasibility signals and require staged clinical evaluation and governance for safe translation. A domain-based qualitative risk of bias and applicability appraisal table is provided in Supplementary File S3.

### 2.6. Declaration of GenAI Tool Usage

Artificial intelligence tools were employed in the generation of figure files and the graphical abstract (Gemini 3.1 Pro). In addition, AI-assisted language editing tools were used to review spelling and formatting. All scientific content, interpretations, and conclusions were developed and verified by the authors, who assume full responsibility for the manuscript.

## 3. The Operator Gap in the POCUS Era

### 3.1. Acquisition as the Limiting Step for Novices

For many novice operators, a dominant failure mode is inability to obtain a clear image rather than incorrect interpretation of an adequate image. Acquisition requires continuous adjustment to patient anatomy, body habitus, and pathology. Learning curves vary substantially across learners and settings [5]. Interpretation and clinical integration remain critical; acquisition support should be viewed as complementary to interpretation training and diagnostic decision support.

### 3.2. Safety Relevance of Unsupervised Acquisition

Error analyses in emergency ultrasonography highlight that incorrect probe position, incomplete protocols, and failure to recognize artifacts contribute to misdiagnosis and inappropriate management [6]. When learners train without structured feedback, acquisition errors can become motor habits that persist despite later instruction.

### 3.3. Implications for Technology and Governance

If acquisition is a dominant bottleneck, then acquisition guidance and automated QA are direct targets for intervention. These functions operate upstream of interpretation and determine whether downstream clinical reasoning is possible.

## 4. The Learning Ecosystem: FOAMed, Informal Learning, and Quality Governance

### 4.1. FOAMed as a Structural Response to Mentorship Scarcity

FOAMed is a global educational movement, particularly in emergency medicine and critical care [9,10]. Its strengths include accessibility, speed, and reach. In ultrasound education, FOAMed can provide cognitive scaffolding such as probe orientation principles and protocol mnemonics. It rarely provides individualized feedback on probe motion or image adequacy.

### 4.2. Quality Heterogeneity and Risks of Habit Fixation

Evaluations of widely available online video-based instructional materials for ultrasound examinations and ultrasound-guided procedures (e.g., YouTube content) show variable quality and incomplete coverage of key steps [15,36,37,38]. When video-based learning becomes a primary modality, learners may practice suboptimal acquisition patterns without corrective feedback. Our concern pertains to uncurated use of heterogeneous resources; high-quality FOAMed content exists and may be valuable when appraised and integrated within structured curricula.

### 4.3. Appraisal and Curation Tools for Online Educational Resources

METRIQ and rMETRIQ support structured appraisal of online educational resources [11,12]. AIR uses expert review and a scoring instrument to curate online resources for trainees, and the revised AIR score improves reliability of appraisal [13,14].

### 4.4. Linking Education to Clinical Governance

Training ecosystems are hybrid, combining local supervision, simulation, and online resources. Governance therefore requires explicit policies on acceptable learning resources, competency assessment, documentation standards, and ongoing QA (Table 1).

## 5. Artificial Intelligence-Assisted Image Acquisition: Functions, Evidence, and Limitations

### 5.1. Functional Taxonomy for Acquisition-Focused Systems

**Figure 3:**
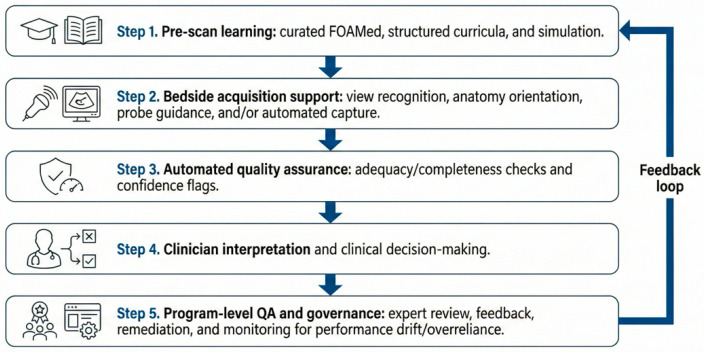
Conceptual workflow of acquisition-focused AI across the point-of-care ultrasound process. The diagram summarizes how AI can support bedside acquisition and automated quality checks while linking clinician interpretation and program-level expert review within a feedback loop.

Acquisition-focused AI functions can be grouped into view recognition, anatomy labeling for orientation, prescriptive probe guidance, automated capture, and post-scan feedback analytics. A functional taxonomy that also includes automated QA and program-level workflow support is summarized in Table 2. A conceptual end-to-end workflow is summarized in Figure 3.

Clinically, acquisition guidance systems combine real-time view recognition and quality feedback with simple, prescriptive cues (or automated capture triggers) that help an operator converge on a target view. These components are commonly implemented with deep learning models, but for clinical readers and implementers the most important details are the intended use (exam scope and outputs), the user training dose, the latency and interface of the feedback, and the system’s failure handling (e.g., low-confidence outputs and escalation to expert review) [28,30].

Prescriptive guidance depends on accurate view recognition, clear cue logic, and robust capture criteria. Studies should report cue logic, latency, and confidence thresholds because these define the intervention experienced by users and influence overreliance risk [20,22,23,30]. Even when diagnostic adequacy is achieved, supported examinations are typically narrow and should be stated explicitly in the intended use statement [28,32].

### 5.2. Clinical Evidence Clusters

Cardiac ultrasound has led clinical evaluation because standard views are well defined and core outcomes are clinically meaningful. Prospective studies show that AI guidance can help novices to acquire echocardiographic views of diagnostic quality for limited clinical parameters [20,21,22]. More recent randomized controlled trials further support measurable gains in acquisition speed and/or image adequacy for novice echocardiography learners using real-time AI guidance [44,45]. A deep-learning prototype has also enabled novices to obtain diagnostic-quality urinary system abdominal ultrasound images in prospective testing [46]. In trauma protocols, AI guidance has been associated with improved acquisition quality for novice operators performing focused assessment with sonography in trauma [23]. In abdominal aortic aneurysm screening, deep learning guidance has been prospectively evaluated for novice operators with image quality assessed by masked expert reviewers [24]. Lung ultrasound guidance has been evaluated in a multicenter validation study and suggests feasibility beyond cardiac and trauma protocols [25]. Table 3 provides an acquisition guidance-focused summary of key prospective or comparative clinical evaluations of AI-guided acquisition and their limitations for translation. Most studies use expert-defined image adequacy endpoints and short training doses; improvements in acquisition quality should not be interpreted as evidence of improved patient outcomes without comparative clinical trials.

Across prospective studies, the most defensible primary endpoint is expert-defined diagnostic adequacy with masked review, complemented by time to view attainment, protocol completeness, and need for expert rescue [20,21,22,23,24,25]. Reporting should specify operator baseline, training dose, and whether feedback is continuous during scanning or limited to capture events, because these factors influence skill transfer beyond the index encounter [5,30].

Overall, the evidence base is currently most mature for real-time guidance and automated capture in constrained cardiac view acquisition, where multiple prospective studies demonstrate improvements in expert-rated image adequacy among novices. In contrast, evidence for automated QA scoring, program-level triage, and generalizable multi-device deployment remains early-stage (Table 4). Future comparative and pragmatic trials should evaluate downstream diagnostic accuracy, clinical decision-making, and patient outcomes, alongside human factors such as overreliance/automation bias, workflow impact, and cost-effectiveness.

### 5.3. Lung Ultrasound Guidance as a Task-Constrained Acquisition Use Case

Lung ultrasound is increasingly used for dyspnea evaluation, but is sensitive to technique and protocol adherence. A multicenter validation study evaluated AI guidance that directed acquisition and automated capture using an eight-zone lung ultrasound protocol performed by trained health care professionals without significant lung ultrasound experience [25]. Clinical translation requires governance and monitoring for performance drift across settings.

Lung ultrasound is well suited to acquisition guidance because protocols are standardized and image interpretation often relies on reproducible artifacts. Guidance systems should enforce zone coverage and probe orientation to avoid false reassurance from incomplete sampling [25]. For acute care programs, defining when lung ultrasound complements computed tomography and when escalation is required can prevent scope creep and align bedside practice with institutional standards [40].

### 5.4. Evidence Gaps and Threats to Generalizability

External validation across devices, sites, and patient populations remains limited in many imaging AI studies, and performance can deteriorate in new settings [41]. In addition, comparative evaluations often have design and reporting limitations that may inflate performance claims, underscoring the need for rigorous prospective assessment [42]. Broader meta-analyses of deep learning diagnostic accuracy in medical imaging demonstrate heterogeneous performance and substantial between-study variability [47]. Outcome heterogeneity is substantial, including variable quality scoring systems and reference standards. Workflow integration and human factors determine effectiveness because prompts, capture logic, and failure handling define the intervention experienced by users [30].

Generalizability is threatened by device-specific integration, differences in presets, and variation in patient body habitus. Studies should describe the spectrum of patients, devices, and acquisition conditions, and include external validation wherever feasible [41,42,48]. Imaging-specific transparency checklists and diagnostic accuracy guidance can strengthen reporting of dataset provenance, reference standards, and bias risk [32,34,35].

## 6. Automated Quality Assurance: From Real-Time Adequacy to Program Governance

### 6.1. Why Quality Assurance Is Required for Scale

Training alone is insufficient when POCUS becomes widely distributed. Governance frameworks and consensus statements emphasize that ongoing QA is required to ensure that acquisition and interpretation remain within acceptable standards and documentation practices [8,39,40]. Quality assurance programs often depend on expert overreads and feedback loops, which become difficult to scale as scan volume increases.

Quality assurance in point-of-care ultrasound programs includes image review against predefined standards, feedback to operators, and documentation that links images to clinical decisions. Consensus recommendations emphasize governance structures that define scope of practice, supervision pathways, and archiving for review [8,39,40]. When acquisition assistance is used, the quality assurance plan should clarify how automated assessments are audited and how discrepancies trigger expert review, because accountability remains clinical rather than technological [30,49].

Automated quality metrics can support competency assessment by quantifying protocol completeness, view diversity, and frequency of nondiagnostic studies over time [26]. Recent proof-of-concept studies have operationalized adequacy definitions into AI-based grading systems for FAST examinations and interpretable echocardiography QC pipelines, suggesting the feasibility of automating aspects of expert review and feedback loops [50,51]. These longitudinal signals can identify skill drift and inform targeted remediation, but they require stable definitions of adequacy and periodic recalibration when devices, presets, or patient populations change [41,48].

### 6.2. Automated Classification and Workflow Support

Automated image classification approaches have been developed as components of QA pipelines in POCUS programs, illustrating feasibility of triage and monitoring workflows [26]. However, prospective patient-facing evaluations of program-level automated QA remain limited. Such approaches can reduce manual sorting and speed feedback, but require external validation, monitoring, and clear escalation thresholds. Given limited prospective evidence for program-level automated QA, early implementations should use conservative thresholds, explicit escalation to expert review, and ongoing monitoring for drift.

From an implementation perspective, automated triage should be designed to minimize unsafe false negative results. Conservative thresholds, clear flags for low-confidence outputs, and a pathway for rapid expert overread are pragmatic safeguards during early deployment [30]. Reporting should specify where the algorithm runs, what data are stored, and how performance is monitored, consistent with reporting extensions for artificial intelligence interventions and diagnostic accuracy studies [28,29,32,33,34].

Technically, automated quality assurance can be implemented as supervised classification or regression models trained on expert-labeled adequacy criteria [52,53,54]. These supervised pipelines can be complemented by unsupervised detection of atypical scans using reconstruction or memory-based embeddings [55]. Separate pipelines can target equipment-level quality using phantom acquisitions to reduce evaluator subjectivity and support maintenance decisions [56]. Audit systems have also been associated with improved acquisition quality after feedback cycles, suggesting a bridge between program governance and individual coaching [57]. Recent proof-of-concept reports have also described guided acquisition tools that provide real-time quality feedback in echocardiography [58].

Program-level review can use sampling strategies that balance feasibility and safety. A proportion of studies should undergo blinded expert review to estimate false negative rates of automated screening, and disagreements should be categorized as acquisition failure or interpretation error to guide remediation [6,35]. When used for research evaluation, these reviews should report reference standards and workflow context in line with early clinical evaluation guidance [30].

## 7. Integrating FOAMed, Simulation, and Artificial Intelligence into a Unified Training Pathway

### 7.1. A Practical Hybrid Model

A pragmatic training pathway can be conceptualized as an integrated pathway (Figure 1) with four functional layers, in which program-level QA is operationalized through automated quality checks and expert feedback. Curated online resources appraised with METRIQ, rMETRIQ, or AIR provide cognitive scaffolding [11,12,13,14]. Simulation supports deliberate practice of probe handling and protocol completion [59]. AI-assisted acquisition guidance provides early technique standardization and immediate feedback during bedside scanning [20,21,22,23,24,25]. Program-level QA and expert feedback support progressive independence and identify skill drift. These components function iteratively, with QA and expert feedback informing targeted remediation and ongoing resource curation.

In this hybrid model, curated online resources provide cognitive framing while deliberate practice builds psychomotor competence. Appraisal tools such as the Medical Education Translational Resources Impact and Quality score and the Approved Instructional Resources framework can select resources that are accurate, complete, and aligned with local protocols [11,12,13,14]. Where video-based learning is common, explicit curation mitigates the risk of adopting incomplete technique described in quality evaluations [15].

Simulation supports repetition without patient risk and can standardize exposure to key views and common artifacts before bedside scanning [59]. Acquisition guidance can then function as just-in-time feedback, reinforcing probe positioning and protocol completion during early independent scans [20,22,23,25]. Programs should specify when guidance is required, when users can override prompts, and how competence is documented once guidance is no longer needed.

Digital mentor functions can deliver just-in-time feedback by combining acquisition and quality-assessment signals with adaptive coaching logic. A mentor can estimate learner proficiency from longitudinal quality trajectories, suggest the next practice task, and taper feedback intensity to avoid cognitive overload. Adaptive feedback policies have been proposed that optimize coaching based on competence and retention while balancing reinforcement of core skills with introduction of new tasks [60,61,62]. In clinical settings, the feedback logic should remain transparent and auditable, and it must be paired with safeguards against overreliance and clear escalation when confidence is low or findings are discordant [30,49].

### 7.2. Institutional Governance and Quality Processes

Governance frameworks for bedside ultrasound emphasize training pathways, documentation, and quality processes. Echocardiography laboratory recommendations and intensive care consensus statements describe institutional responsibilities for training and for maintaining quality standards [8,39,40]. Acquisition guidance and automated QA should be implemented within these governance structures rather than as standalone tools.

Institutional governance is needed to prevent overreliance and to define escalation pathways. Recommendations for echocardiography laboratories and intensive care ultrasound emphasize defined scope, supervision, and ongoing review [8,39,40]. Embedding acquisition assistance within these structures supports safe scaling and clarifies that the technology augments training rather than replaces expert judgment [18,19,49].

Competency progression should not rely solely on scan counts. Learning curves vary, and programs can combine supervised milestones, structured image portfolios, and periodic objective assessments to decide when trainees can practice independently [5]. When digital resources and acquisition guidance are used, maintaining a feedback loop that links performance data to targeted coaching can preserve clinical reasoning skills and reduce overconfidence during transitions to autonomy [18,30].

## 8. Implementation, Ethics, and Reporting Transparency

### 8.1. Data Quality and Preparation

Robust development depends on representative datasets, consistent labeling, and transparent preprocessing. Data preparation choices influence performance and reproducibility [48]. Ultrasound adds complexity because acquisition is part of the signal. Models trained on expert images may not generalize to novice acquisition unless novice data are represented.

### 8.2. External Validation and Domain Shift

External validation is a requirement for clinical trust. A systematic review of external validation of deep learning algorithms in radiologic diagnosis highlights frequent absence of robust validation and variable performance in new settings [41].

### 8.3. Ethical and Equity Considerations

AI systems can amplify disparities when trained on nonrepresentative data or when proxy outcomes are poorly specified. Bias in widely used health management algorithms illustrates how inequities can arise even when models appear accurate at the population level [63]. Although not imaging-specific, this example highlights how proxy outcomes and dataset shift can create inequitable performance; analogous risks exist in ultrasound AI when training data underrepresent certain patient groups, devices, or acquisition styles. Implementation therefore requires subgroup performance reporting when feasible, clarity about accountability, and post-deployment monitoring [41,49]. For deployment, governance plans should address data stewardship and privacy, clarify regulatory classification (e.g., regulated medical device software vs decision support), and define accountability and escalation pathways when AI-guided acquisition fails, confidence is low, or clinical findings are discordant.

### 8.4. Reporting Guidance as a Translation Tool

Transparent reporting improves interpretability and supports replication. CONSORT-AI and SPIRIT-AI address reporting for trials and protocols involving AI interventions [28,29]. DECIDE-AI provides a framework for early clinical evaluation and emphasizes workflow integration and human factors [30]. TRIPOD+AI supports transparency for prediction models, and STARD 2015 and STARD-AI support diagnostic accuracy reporting [31,32,33]. CLAIM provides an imaging-specific checklist for dataset provenance, labeling, and methodological transparency [34].

## 9. Future Directions and Research Agenda

### 9.1. Standardized Outcomes for Acquisition and Quality Assurance

Future evaluations should converge on standardized definitions of diagnostic adequacy, protocol completeness, and quality scoring. Without standardized outcomes, synthesis remains limited and device comparisons are difficult.

### 9.2. Positioning This Review Within Existing Literature

**Figure 4:**
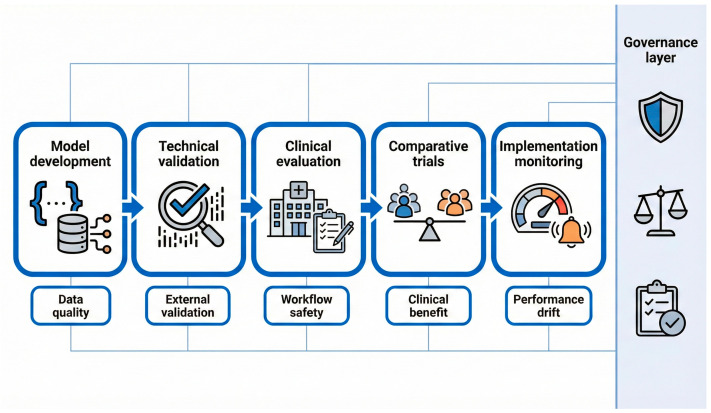
Evidence-to-implementation pathway for acquisition-focused artificial intelligence and automated quality assurance in point-of-care ultrasound (conceptual framework). Shows a staged pathway for acquisition-focused AI and automated QA: model development, technical validation, clinical evaluation, comparative trials, and implementation monitoring. Each stage emphasizes a core focus (data quality, external validation, workflow safety, clinical benefit, and performance drift) under a cross-cutting governance layer for accountability and oversight. This framework is conceptual and intended to support staged evaluation and safe translation.

Recent reviews have summarized broad AI applications in POCUS across interpretation, education, workflow automation, and quality assurance [64]. This review focuses on acquisition and QA as upstream determinants of safety and scalability, and integrates these functions with the contemporary learning ecosystem. To operationalize this focus, we provide a function-based taxonomy (Table 2) and an evidence-to-implementation pathway (Figure 4) that link bedside acquisition support to scalable QA and clinical governance.

### 9.3. A Minimum Evidence Pathway for Clinical Translation

Safe translation of acquisition-focused AI and automated QA requires staged evidence generation rather than isolated feasibility reports. We propose an evidence-to-implementation pathway that sequences model development, technical validation, clinical evaluation, comparative trials, and implementation monitoring, and pairs each stage with core evaluation foci and governance considerations (Figure 4). Early clinical evaluation should describe intended use, workflow integration, user training, and failure handling in line with DECIDE-AI [30]. Comparative evaluations should use CONSORT-AI and SPIRIT-AI when randomized designs or protocol-driven trials are conducted [28,29]. Studies that function as diagnostic accuracy evaluations should report reference standards and cohort spectrum in line with STARD 2015 and STARD-AI [32,33]. Prediction model studies should follow TRIPOD+AI [31]. Imaging-specific transparency items such as dataset provenance, labeling procedures, and missing data handling should be documented using CLAIM where applicable [34].

### 9.4. Limitations

This narrative review provides a qualitative synthesis and does not generate primary patient data. Although we used explicit scope definitions and structured database searches, we did not conduct a full systematic review workflow or meta-analysis, and we did not calculate a formal quantitative risk-of-bias score; instead, we provided a domain-based qualitative appraisal table (informed by QUADAS-2) in Supplementary File S3. Accordingly, evidence capture and weighting may be incomplete or subjective. Our focus on acquisition guidance, automated quality assurance (QA), and the FOAMed-influenced learning ecosystem may underrepresent interpretive AI and downstream diagnostic performance. The available clinical literature remains early-phase and heterogeneous, often device- or protocol-constrained, with limited external validation and few patient-centered outcomes; thus, our findings should be interpreted as feasibility signals rather than definitive evidence of effectiveness. We did not directly review FOAMed posts or videos and instead relied on peer-reviewed evaluations and appraisal tools, which limits inference about the breadth and quality of real-world online content. Finally, the proposed integrated pathway and evidence-to-implementation framework are conceptual and hypothesis-generating and require prospective validation, local adaptation, and post-deployment monitoring for safety, equity, and performance drift. We did not preregister a protocol or apply a uniform appraisal instrument across all study designs; consequently, conclusions should be interpreted cautiously [27,41,42].

We also prioritized peer-reviewed journal articles and may underrepresent conference proceedings and proprietary evaluations in this rapidly evolving domain. Many reports provide incomplete technical details, including model architecture, training data composition, whether networks were fully trained or fine-tuned, and deployment constraints, which limit reproducibility and engineering assessment [65,66,67]. Additionally, the empirical evidence for acquisition mentoring remains early phase. Many studies are single-center and rely on surrogate endpoints such as time to target view and expert-rated adequacy rather than patient outcomes or cost-effectiveness. External validation across devices is uncommon, and performance may degrade under dataset shift, underscoring the need for device-aware quality monitoring and periodic recalibration [41,48,56,57]. Moreover, our focus on the pre-diagnostic phase may underemphasize downstream interpretation models, even though acquisition and interpretation are increasingly integrated in commercial workflows.

## 10. Conclusions

POCUS has transformed bedside diagnostics, but operator dependence remains a primary barrier to safe scale. Informal self-directed learning, including FOAMed resources, can help address training gaps but requires structured appraisal and curation. Acquisition-focused AI targets an upstream bottleneck by helping clinicians obtain adequate images and supporting scalable feedback loops. Prospective evidence across cardiac ultrasound, trauma protocols, abdominal aortic aneurysm screening, and lung ultrasound supports the feasibility of AI-guided acquisition for constrained tasks, primarily measured by expert-defined image adequacy. Future work should test whether these improvements translate into patient-centered outcomes (e.g., diagnostic accuracy, time to treatment, morbidity/mortality) and should evaluate workflow efficiency and cost-effectiveness. Translation into routine practice will require external validation, standardized outcomes, transparent reporting, and governance structures that integrate training with continuous QA.

## Figures and Tables

**Table 1:** Educational ecosystem and governance tools relevant to ultrasound training. Maps three scale-up risks (inconsistent FOAMed quality, device diffusion without mentorship, and AI-guided acquisition hazards such as overreliance and domain shift) to governance responses, including curated resource appraisal (METRIQ/rMETRIQ/AIR), structured curricula and documentation standards, QA programs with feedback loops, and technical safeguards such as external validation and monitoring.

Domain	Primary Risk	Governance Tools
Informal unsupervised learning	Variable content quality and habit fixation risk [15]	METRIQ, rMETRIQ, AIR [11,12,13,14]
Device diffusion without mentorship	Skill variability and inconsistent QA [5,6,7]	Curricula and QA programs [7,8,39,40]
AI-guided acquisition	Overreliance and domain shift [41,42]	External validation and transparent reporting [28,29,30,31,32,33,34]

Abbreviations: AIR, Approved Instructional Resources; FOAMed, free open access medical education; METRIQ, Medical Education Translational Resources Impact and Quality; QA, quality assurance; rMETRIQ, Revised Medical Education Translational Resources Impact and Quality.

**Table 2:** Taxonomy of acquisition-focused artificial intelligence and automated quality assurance for ultrasound. Summarizes acquisition-focused AI functions and how they support bedside scanning or QA workflows: view recognition, anatomy labeling for orientation, prescriptive probe guidance, automated capture, post-scan feedback analytics, automated quality scoring, and program-level QA workflow support (triage/monitoring). Example endpoints are illustrative and should align with intended use, reference standards, and downstream decisions.

Function Group	Workflow Role	Example Endpoints
View recognition	Confirms target views are obtained [43]	View attainment rate, time to view
Anatomy labeling for orientation	Highlights structures and landmarks to support probe orientation [16,17]	Label accuracy, time to correct orientation
Prescriptive probe guidance	Guides probe motion toward target view [20,21,22,23,24,25]	Diagnostic adequacy by experts, completion time
Automated capture	Records when criteria are met	Clip quality, protocol completeness
Post-scan feedback analytics	Provides post-scan feedback on acquisition errors and protocol gaps	Error detection rate, subsequent scan quality improvement
Automated quality assurance	Scores adequacy and completeness	Adequacy sensitivity and specificity, triage performance
Program-level QA workflow support	Reduces expert review burden [26]	Review workload, feedback timeliness

Abbreviations: AI, artificial intelligence; QA, quality assurance.

**Table 3:** Acquisition guidance-focused summary of clinical evidence for artificial intelligence-assisted acquisition guidance in point-of-care ultrasound. Summarizes key prospective or comparative evaluations of AI-guided POCUS acquisition by clinical domain and function. “Design and key findings” describes study design and primary acquisition-quality outcomes (expert-defined). “Key limitations” notes generalizability threats (single site/device, lab context, limited spectrum) and needs for external validation and implementation evaluation.

Study	Clinical Domain	AI Function Focus	Design and Key Findings	Key Limitations for Translation
Narang et al. 2021 [20]	Transthoracic echocardiography	Guidance with automated left ventricular ejection fraction output	Prospective diagnostic study. Nurses without prior ultrasonography experience scanned 240 patients. Diagnostic quality was high for prespecified targets compared with expert scans.	Defined target exam and specific device. External generalizability requires multicenter and multi-device validation.
Schneider et al. 2021 [21]	Echocardiography acquisition	Real-time guidance for standard views	Comparative clinical study. Medical students performed 57 patient scans after brief training. Automated outputs aligned with expert measurements when image quality was adequate.	Pilot scale. Performance depends on diagnostic quality images and broader validation is needed.
Mor-Avi et al. 2023 [22]	Cardiac point-of-care ultrasound	Real-time quality feedback	Prospective multicenter study. Nurses and residents with minimal training scanned 240 patients. Novice scans were frequently sufficient for core cardiac targets.	Device and workflow specific. Reporting of training dose and failure handling remains critical.
Kumar et al. 2025 [44]	Limited echocardiography acquisition	Deep-learning guidance for limited echocardiogram acquisition	Randomized controlled trial. Novice clinicians acquired limited echocardiograms with deep-learning guidance. Guidance improved acquisition speed and/or expert-rated image adequacy.	Defined exam scope and specific implementation. Generalizability across devices/sites and downstream clinical outcomes remain to be established.
Karni et al. 2025 [45]	Cardiac ultrasound (apical views)	AI-enhanced real-time guidance for apical view acquisition	Educational study. AI-enhanced guidance improved novices’ apical 4-chamber and apical 5-chamber view acquisition.	Education-focused endpoints. External validation across patient spectra, devices, and clinical settings is needed.
Chiu et al. 2023 [23]	Focused assessment with sonography in trauma	Real-time AI guidance and quality feedback for FAST (Morison pouch) view acquisition	Randomized quality improvement study in a laboratory context. Thirty ultrasound-naïve operators (RNs/NPs/EMTs) performed FAST (Morison pouch) scanning on healthy volunteers; AI guidance increased diagnostic-quality scores and the rate of acceptable clips, with slightly longer completion time early in training.	Laboratory environment. Clinical trauma validation and patient-centered outcomes remain needed.
Chiu et al. 2024 [24]	Abdominal aortic aneurysm screening	Guidance with automated capture and annotation	Prospective outpatient evaluation. Nurses without prior ultrasonography experience scanned 184 patients. Image quality by masked experts approached physician scans in the study context.	Low disease prevalence and single-center setting. Replication and implementation evaluation are required.
Ossaba et al. 2024 [46]	Urinary system abdominal ultrasound	Deep-learning modular prototype for acquisition guidance	Prospective validation study. Prototype guidance enabled novices to obtain diagnostic-quality urinary system ultrasound images.	Prototype/early-phase evidence. Broader generalizability and implementation evaluation remain limited.
Baloescu et al. 2025 [25]	Lung ultrasound for dyspnea	Real-time acquisition guidance with automated capture and B-line annotation (eight-zone lung ultrasound protocol)	Multicenter diagnostic validation study. Adults with shortness of breath underwent two lung ultrasound examinations (trained health care professionals using Lung Guidance AI vs a lung ultrasound expert without AI); masked expert readers assessed diagnostic quality. With AI guidance, 98.3% of nonexpert-acquired studies met diagnostic-quality standards and were not statistically different from expert-acquired studies.	Protocol constrained. Governance is needed to address overreliance and performance drift.

Abbreviations: AI, artificial intelligence; FAST, focused assessment with sonography in trauma; POCUS, point-of-care ultrasound.

**Table 4:** Evidence maturity matrix for acquisition-focused artificial intelligence and automated quality assurance in point-of-care ultrasound. Qualitative summary of evidence maturity by function and evaluation stage; ratings are narrative (not formal GRADE).

Function/Use Case	Technical Validation	Prospective Clinical Evaluation	Comparative Evaluation vs. Standard Practice	Patient-Centered Outcomes	Implementation/Monitoring
Real-time guidance + automated capture (cardiac views)	Moderate	Moderate	Moderate	Not yet	Emerging
Real-time guidance (FAST, AAA, lung protocols)	Emerging	Emerging	Limited	Not yet	Not yet
View recognition/anatomy labeling (as components)	Moderate	Indirect	Indirect	Not yet	Not yet
Automated quality scoring (real-time or post-scan)	Emerging	Limited	Not yet	Not yet	Emerging
Program-level QA triage/workflow analytics	Limited	Limited	Not yet	Not yet	Emerging
Post-scan feedback analytics for skill drift/remediation	Emerging	Not yet	Not yet	Not yet	Not yet

Abbreviations: AAA, abdominal aortic aneurysm; FAST, focused assessment with sonography in trauma; QA, quality assurance. Note: evidence maturity ratings are qualitative and reflect the stage and breadth of published evaluations captured in this review; most studies report surrogate endpoints (e.g., expert-rated image adequacy) rather than patient outcomes.

## Data Availability

This article is a narrative review based exclusively on previously published literature. No new data were created or analyzed; therefore, data sharing is not applicable.

## Supplementary Materials

The following supporting information can be downloaded at https://www.mdpi.com/article/10.3390/diagnostics16060858/s1, Supplementary File S1: Full database search strategies for MEDLINE, Embase, Scopus, and Web of Science, including date limits and field tags; Supplementary File S2: SANRA checklist; Supplementary File S3: Qualitative assessment of study limitations, bias risk, and generalizability.
