# Supplementary material for: Beyond Computer-Aided Diagnosis: Artificial Intelligence as a “Digital Mentor” for POCUS Image Acquisition and Quality Assurance: A Narrative Review
Source: Diagnostics (Basel). 2026 Mar 13;16(6):858. doi: 10.3390/diagnostics16060858 (PMC13025692; doi:10.3390/diagnostics16060858)
Supplement: Supplementary file 1 [file diagnostics-16-00858-s001.zip › diagnostics-4175903-supplementary.pdf]

Supplementary File S1. Full database search strategies

This file provides reproducible search strategies for the narrative review. Two concept sets were searched: Search A for artificial intelligence-assisted ultrasound image acquisition and automated quality assurance; Search B for free open access medical education and online ultrasound learning quality appraisal.

Summary of database yields at search time: Search A total n = 6,252; Search B total n = 7,537; combined n = 13,789 (before deduplication and screening).

Search A. Artificial intelligence assisted image acquisition and automated quality assurance

MEDLINE

Platform: PubMed

Date searched: February 23, 2026

Retrieved record count: n = 737

Limits: English; no date restriction; Humans filter applied where supported

Notes: Use [tiab] fields for title and abstract keywords. The MeSH component is included to improve recall for indexed records. Apply PubMed filters for English and Humans rather than adding population terms to the search string.

Full search string:

```
(
 (
  ("point-of-care ultrasound"[tiab] OR "point of care ultrasound"[tiab] OR pocus[tiab] OR
"bedside ultrasound"[tiab]
   OR ultrasound[tiab] OR ultrasonograph*[tiab] OR sonograph*[tiab] OR
echocardiograph*[tiab] OR "lung ultrasound"[tiab]
   OR "focused assessment with sonography in trauma"[tiab] OR "FAST exam"[tiab] OR
"FAST scan"[tiab] OR "FAST protocol"[tiab])
  AND
  ("artificial intelligence"[tiab] OR "machine learning"[tiab] OR "deep learning"[tiab] OR
"computer vision"[tiab]
   OR "neural network"[tiab] OR "neural networks"[tiab]
   OR "convolutional neural network"[tiab] OR "convolutional neural networks"[tiab])
  AND
```

```
  ("image acquisition"[tiab] OR acquisition[tiab]
   OR "probe guidance"[tiab] OR "probe position*"[tiab] OR "probe movement"[tiab] OR "probe motion"[tiab]
   OR "scan guidance"[tiab] OR "scanning guidance"[tiab]
   OR "view recognition"[tiab] OR "view classification"[tiab] OR "view identification"[tiab]
   OR "standard view*"[tiab] OR "standard plane*"[tiab] OR "plane detection"[tiab]
   OR "anatomy label*"[tiab] OR "anatomic label*"[tiab]
   OR "auto capture"[tiab] OR "automated capture"[tiab]
   OR "quality assurance"[tiab] OR "quality assessment"[tiab] OR "quality control"[tiab]
   OR "image quality"[tiab] OR adequacy[tiab]
   OR "protocol adherence"[tiab] OR "protocol completeness"[tiab])
 )
 OR
 (
  ("Ultrasonography"[Mesh] OR "Echocardiography"[Mesh])
  AND
  ("Artificial Intelligence"[Mesh] OR "Machine Learning"[Mesh])
  AND
  ("image acquisition"[tiab] OR acquisition[tiab]
   OR "probe guidance"[tiab]
   OR "view recognition"[tiab] OR "view classification"[tiab]
   OR "quality assurance"[tiab] OR "quality assessment"[tiab] OR "image quality"[tiab] OR adequacy[tiab])
 )
)
```

Embase

Platform: Ovid Embase

Date searched: February 23, 2026

Retrieved record count: n = 1873

Limits: English; no date restriction; Human and English limits applied

Notes: Emtree explosion terms are used where supported. Field tags are shown as .ti,ab. and limits include human and English language.

Full search string:

```
(
  ("point-of-care ultrasound" OR "point of care ultrasound" OR pocus OR "bedside
ultrasound"
   OR ultrasound OR ultrasonograph* OR sonograph* OR echocardiograph* OR "lung
ultrasound"
   OR "focused assessment with sonography in trauma" OR "FAST exam" OR "FAST scan"
OR "FAST protocol").ti,ab.
  OR exp ultrasonography/
  OR exp echocardiography/
)
AND
(
  exp artificial intelligence/
  OR exp machine learning/
  OR exp deep learning/
  OR exp neural network/
  OR ("artificial intelligence" OR "machine learning" OR "deep learning" OR "computer
vision"
     OR neural network* OR convolutional neural network*).ti,ab.
)
AND
(
  ("image acquisition" OR acquisition
   OR "probe guidance" OR "probe position*" OR "probe movement" OR "probe motion"
   OR "scan guidance" OR "scanning guidance"
   OR "view recognition" OR "view classification" OR "view identification"
   OR "standard view*" OR "standard plane*" OR "plane detection"
   OR "anatomy label*" OR "anatomic label*"
   OR "auto capture" OR "automated capture"
   OR "quality assurance" OR "quality assessment" OR "quality control"
   OR "image quality" OR adequacy
   OR "protocol adherence" OR "protocol completeness").ti,ab.
)
limit to (human and english language)
```

Scopus

Platform: Elsevier Scopus

Date searched: February 23, 2026

Retrieved record count: n = 2019

Limits: English; no date restriction

Notes: Use TITLE-ABS-KEY. Apply document type filters according to journal requirements if needed.

Full search string:

```
TITLE-ABS-KEY (
  "point-of-care ultrasound" OR "point of care ultrasound" OR POCUS OR "bedside ultrasound"
  OR ultrasound OR ultrasonograph* OR sonograph* OR echocardiograph* OR "lung ultrasound"
  OR "focused assessment with sonography in trauma" OR "FAST exam" OR "FAST scan" OR "FAST protocol"
)
AND TITLE-ABS-KEY (
  "artificial intelligence" OR "machine learning" OR "deep learning" OR "computer vision"
  OR "neural network*" OR "convolutional neural network*"
)
AND TITLE-ABS-KEY (
  "image acquisition" OR acquisition
  OR "probe guidance" OR "probe position*" OR "probe movement" OR "probe motion"
  OR "scan guidance" OR "scanning guidance"
  OR "view recognition" OR "view classification" OR "view identification"
  OR "standard view*" OR "standard plane*" OR "plane detection"
  OR "anatomy label*" OR "anatomic label*"
  OR "auto capture" OR "automated capture"
  OR "quality assurance" OR "quality assessment" OR "quality control"
  OR "image quality" OR adequacy
  OR "protocol adherence" OR "protocol completeness"
)
```

Web of Science Core Collection

Platform: Clarivate Web of Science

Date searched: February 23, 2026

Retrieved record count: n = 1623

Limits: English; no date restriction

Notes: Use TS for topic search. Apply refinement for document types if needed.

Full search string:

TS=(("point-of-care ultrasound" OR "point of care ultrasound" OR POCUS OR "bedside ultrasound" OR ultrasound OR ultrasonograph* OR sonograph* OR echocardiograph* OR "lung ultrasound" OR "focused assessment with sonography in trauma" OR "FAST exam" OR "FAST scan" OR "FAST protocol") AND ("artificial intelligence" OR "machine learning" OR "deep learning" OR "computer vision" OR "neural network*" OR "convolutional neural network*") AND ("image acquisition" OR acquisition OR "probe guidance" OR "probe position*" OR "probe movement" OR "probe motion" OR "scan guidance" OR "scanning guidance" OR "view recognition" OR "view classification" OR "view identification" OR "standard view*" OR "standard plane*" OR "plane detection" OR "anatomy label*" OR "anatomic label*" OR "auto capture" OR "automated capture" OR "quality assurance" OR "quality assessment" OR "quality control" OR "image quality" OR adequacy OR "protocol adherence" OR "protocol completeness")) NOT TS=(animal OR animals OR mouse OR mice OR rat OR rats OR rodent* OR porcine OR pig OR pigs OR swine OR bovine OR cow OR cattle OR sheep OR ovine OR dog OR dogs OR canine OR feline OR cat OR cats OR rabbit* OR monkey* OR primate* OR "in vitro" OR phantom*)

Search B. Free open access medical education and online ultrasound learning quality appraisal

Search B was executed in two complementary approaches: (B1) ultrasound-specific FOAMed/online learning and resource quality appraisal; (B2) a targeted tool-focused search for foundational appraisal instruments (METRIQ/rMETRIQ and AIR), which may not include ultrasound terms in the title or abstract.

MEDLINE

Platform: PubMed

Date searched: February 23, 2026

Retrieved record count: n = 944 (B1); 49 (B2)

Limits: English; no date restriction; Humans filter applied where supported

Notes: Search B targets FOAMed and online learning quality appraisal in ultrasound education. Apply PubMed filters for English and Humans.

Full search string (B1, ultrasound-specific):

```
(
  ("point-of-care ultrasound"[tiab] OR "point of care ultrasound"[tiab] OR pocus[tiab]
   OR ultrasound[tiab] OR ultrasonograph*[tiab] OR sonograph*[tiab] OR
echocardiograph*[tiab] OR "lung ultrasound"[tiab])
  AND
  (FOAMed[tiab] OR "free open access medical education"[tiab] OR "open access medical
education"[tiab]
   OR "online learning"[tiab] OR "online education"[tiab] OR "social media"[tiab]
   OR youtube[tiab] OR video*[tiab] OR blog*[tiab] OR podcast*[tiab]
   OR "online educational resource*"[tiab] OR "educational resource*"[tiab])
  AND
  (education[tiab] OR training[tiab] OR curriculum[tiab] OR learning[tiab]
   OR appraisal[tiab] OR "quality appraisal"[tiab] OR "quality assessment"[tiab]
   OR "content quality"[tiab]
   OR METRIQ[tiab] OR rMETRIQ[tiab]
   OR "Approved Instructional Resources"[tiab] OR "Approved Instructional Resources
Series"[tiab] OR "AIR score"[tiab]
   OR "instructional resource*"[tiab])
)
```

Full search string (B2, tool-focused targeted search):

```
(METRIQ[tiab] OR rMETRIQ[tiab] OR "Approved Instructional Resources"[tiab] OR
"Approved Instructional Resources Series"[tiab] OR "AIR score"[tiab])
```

Embase

Platform: Ovid Embase

Date searched: February 23, 2026

Retrieved record count: n = 2716 (B1); 132 (B2)

Limits: English; no date restriction; Human and English limits applied

Notes: Search B uses FOAMed/online learning concepts with ultrasound terms (B1) and an additional targeted tool search (B2). Review limits before execution.

Full search string (B1, ultrasound-specific):

```
(
  ("point-of-care ultrasound" OR "point of care ultrasound" OR pocus
   OR ultrasound OR ultrasonograph* OR sonograph* OR echocardiograph* OR "lung
ultrasound").ti,ab.
  OR exp ultrasonography/
  OR exp echocardiography/
)
AND
(
  (foamed OR "free open access medical education" OR "open access medical education"
   OR "online learning" OR "online education" OR "social media"
   OR youtube OR video* OR blog* OR podcast*
   OR "online educational resource*" OR "educational resource*").ti,ab.
)
AND
(
  (education OR training OR curriculum OR learning
   OR appraisal OR "quality appraisal" OR "quality assessment" OR "content quality"
   OR metriq OR rmetriq
   OR "approved instructional resources" OR "approved instructional resources series" OR
"air score"
   OR "instructional resource*").ti,ab.
)
limit to (human and english language)
```

Full search string (B2, tool-focused targeted search):

```
(metriq OR rmetriq OR "approved instructional resources" OR "approved instructional
resources series" OR "air score").ti,ab.
limit to (human and english language)
```

Scopus

Platform: Elsevier Scopus

Date searched: February 23, 2026

Retrieved record count: n = 1681 (B1); 80 (B2)

Limits: English; no date restriction

Notes: Use TITLE-ABS-KEY. Consider limiting to relevant subject areas if needed.

Full search string (B1, ultrasound-specific):

```
TITLE-ABS-KEY (
  "point-of-care ultrasound" OR "point of care ultrasound" OR POCUS
  OR ultrasound OR ultrasonograph* OR sonograph* OR echocardiograph* OR "lung
ultrasound"
)
AND TITLE-ABS-KEY (
  FOAMed OR "free open access medical education" OR "open access medical education"
  OR "online learning" OR "online education" OR "social media"
  OR youtube OR video* OR blog* OR podcast*
  OR "online educational resource*" OR "educational resource*"
)
AND TITLE-ABS-KEY (
  education OR training OR curriculum OR learning
  OR appraisal OR "quality appraisal" OR "quality assessment" OR "content quality"
  OR METRIQ OR rMETRIQ
  OR "Approved Instructional Resources" OR "Approved Instructional Resources Series" OR
"AIR score"
  OR "instructional resource*"
)
```

Full search string (B2, tool-focused targeted search):

```
TITLE-ABS-KEY ( METRIQ OR rMETRIQ OR "Approved Instructional Resources" OR
"Approved Instructional Resources Series" OR "AIR score" )
```

Web of Science Core Collection

Platform: Clarivate Web of Science

Date searched: February 23, 2026

Retrieved record count: n = 1851 (B1); 84 (B2)

Limits: English; no date restriction

Notes: Use TS for topic search. Consider refining to document types Article and Review if needed.

Full search string (B1, ultrasound-specific):

TS=(("point-of-care ultrasound" OR "point of care ultrasound" OR POCUS OR ultrasound OR ultrasonograph* OR sonograph* OR echocardiograph* OR "lung ultrasound") AND (FOAMed OR "free open access medical education" OR "open access medical education" OR "online learning" OR "online education" OR "social media" OR youtube OR video* OR blog* OR podcast* OR "online educational resource*" OR "educational resource*") AND (education OR training OR curriculum OR learning OR appraisal OR "quality appraisal" OR "quality assessment" OR "content quality" OR METRIQ OR rMETRIQ OR "Approved Instructional Resources" OR "Approved Instructional Resources Series" OR "AIR score" OR "instructional resource*")) NOT TS=(animal OR animals OR mouse OR mice OR rat OR rats OR rodent* OR porcine OR pig OR pigs OR swine OR bovine OR cow OR cattle OR sheep OR ovine OR dog OR dogs OR canine OR feline OR cat OR cats OR rabbit* OR monkey* OR primate* OR "in vitro" OR phantom*)

Full search string (B2, tool-focused targeted search):

TS=(METRIQ OR rMETRIQ OR "Approved Instructional Resources" OR "Approved Instructional Resources Series" OR "AIR score") NOT TS=(animal OR animals OR mouse OR mice OR rat OR rats OR rodent* OR porcine OR pig OR pigs OR swine OR bovine OR cow OR cattle OR sheep OR ovine OR dog OR dogs OR canine OR feline OR cat OR cats OR rabbit* OR monkey* OR primate* OR "in vitro" OR phantom*)

Abbreviations

AIR, Approved Instructional Resources

AI, artificial intelligence

Emtree, Embase subject headings

FOAMed, free open access medical education

MeSH, Medical Subject Headings

METRIQ, Medical Education Translational Resources Impact and Quality

POCUS, point-of-care ultrasound

QA, quality assurance

rMETRIQ, Revised Medical Education Translational Resources Impact and Quality

TIAB, title/abstract field tag in PubMed

TITLE-ABS-KEY, Scopus field tag for title/abstract/keywords

TS, topic field tag in Web of Science

Supplementary File S2

The Scale for the Assessment of Narrative Review Articles (SANRA) checklist was used as a reporting guide. The table below summarizes compliance and manuscript locations.

SANRA item	Score (0-2)	Manuscript location	Notes / compliance statement
1. Justification of the article's importance for the readership	2	Introduction 1.1–1.4; Sections 3–4	Describes the operator gap with handheld POCUS diffusion and rationale for acquisition-focused AI as a scalable support tool.
2. Statement of concrete aims / objectives	2	Abstract; Introduction 1.4; End of Introduction	States the purpose to synthesize acquisition-focused AI and automated QA evidence and propose a practical pathway for training and governance.
3. Description of the literature search	2	Methods 2.2; Supplementary Table S1; Figure 2	Provides databases, search dates, concept sets, limits, and reproducible full search strings; describes screening and selection workflow.
4. Referencing	2	Throughout; Tables 1–4	Uses current and relevant citations with standardized formatting; key evidence summarized in tables.
5. Scientific reasoning	2	Sections 5–9	Links evidence to conceptual pathway; discusses limitations, generalizability, staged evaluation, and governance considerations.
6. Appropriate presentation of data	2	Figures 1–4; Tables 1–4	Uses tables and conceptual figures to summarize taxonomy, evidence clusters, and implementation pathway; avoids duplicating table content in the main text.

Supplementary File S3

A formal quantitative risk-of-bias score was not calculated because included studies were heterogeneous. Instead, the table below summarizes recurring domain-based limitations relevant to risk of bias and applicability (patient spectrum, intervention/comparator definition, reference standard, and flow/timing). This qualitative appraisal focuses on representative early clinical evaluation and QA program studies.

Study	Patient spectrum / selection	Intervention & comparator clarity	Reference standard / blinding	Flow, timing, and missing data	Applicability / generalizability notes
Narang et al 2021 [20]	Single device and predefined target exam; potential selection/spectrum limitation not fully described in this review.	AI-guided acquisition by ultrasound-naïve nurses; comparator to expert scans described.	Expert scans and expert quality/measurement reference; masking reported in study summary.	Large sample; flow across operators/patients may introduce learning-curve effects.	Single-center and device-specific; multi-device, multi-site validation needed.
Schneider et al 2021 [21]	Small pilot cohort; limited spectrum and setting.	Brief training dose; comparator and endpoints rely on adequate images.	Expert measurements used when images adequate; dependence on acquisition quality.	Pilot scale; unclear handling of failed scans.	Generalizability limited; requires broader validation.
Mor-Avi et al 2023 [22]	Multicenter design improves spectrum but still	Minimal training operators; intervention is real-	Masked expert reading noted in summary; reference standard is	Flow/timing details and failure handling	Device/workflow specificity; report confidence thresholds

	device/workflow constrained.	time quality feedback.	image quality/targets.	variably reported.	and escalation pathways.
Chiu et al 2023 [23]	Laboratory study in healthy volunteers; spectrum differs from trauma patients.	Randomized quality improvement; AI guidance vs usual acquisition on volunteers.	Quality scoring by expert readers; applicability to clinical trauma remains uncertain.	Short time horizon; learning curve and longer completion time early.	Needs clinical trauma validation and patient-centered outcomes.
Chiu et al 2024 [24]	Outpatient screening with low disease prevalence; single-center.	Nurse acquisition with AI guidance compared with physician scans.	Masked expert image-quality review described in summary.	Implementation factors and failed scans not detailed here.	Replication across sites/devices and implementation evaluation needed.
Baloescu et al 2025 [25]	Adults with dyspnea; multicenter improves spectrum but protocol constrained.	AI-guided acquisition by trained professionals compared with expert without AI.	Masked expert readers assessed diagnostic quality; strong reference standard for adequacy.	Protocolized 8-zone exam; patient outcomes not assessed.	Generalizability to other protocols/devices and post-deployment drift monitoring needed.
Blaivas et al 2020 [26]	Program-level QA context; case mix and spectrum may vary.	Automated QA/triage within QA workflow; comparator is expert review burden.	Reference standard typically expert review; details depend on local QA implementation.	Feasibility context; limited comparative outcome reporting.	Evidence is early; requires prospective evaluation of workflow, safety, and human factors.
